# Application of LC–MS/MS in the Mycotoxins Studies

**DOI:** 10.3390/toxins12040272

**Published:** 2020-04-23

**Authors:** Laura Gámiz-Gracia, Ana M. García-Campaña, Natalia Arroyo-Manzanares

**Affiliations:** 1 Department of Analytical Chemistry, Faculty of Sciences, University of Granada, 18071 Granada, Spain; amgarcia@ugr.es; 2 Department of Analytical Chemistry, Faculty of Chemistry, University of Murcia, 30100 Murcia, Spain; natalia.arroyo@um.es

Mycotoxins are secondary metabolites produced by fungi of different species (mainly *Aspergillus, Fusarium,* and *Penicillium*) with toxic effects for humans and animals that can contaminate food and feed. Notifications on the Rapid Alert System for Food and Feed (RASFF) concerning mycotoxins are becoming frequent, being among the “top 10” hazards reported on food products, mainly cereals and nuts [1]. The European Union (EU), among others, has established maximum permitted or recommended levels for several mycotoxins, including aflatoxins (AF) B1, B2, G1, G2 and M1, ochratoxin A (OTA), patulin (PAT), deoxynivalenol (DON), zearalenone (ZEN), fumonisins (FB1 and FB2), HT-2 and T-2 toxins, or citrinin (CIT) in different food commodities [2]. However, there are other mycotoxins not included in regulations, the so-called “emerging mycotoxins”, whose toxicity is still not clear, that could also pose a risk to humans and animals. This group includes *Alternaria* toxins, sterigmatocystin (STC), phomopsins, and *Fusarium* toxins as enniatins (ENN) or beauvericin (BEA), among others [3]. Moreover, the so-called “modified or masked mycotoxins” (produced as a consequence of a detoxification strategy of the host plant of the fungus or during food processing) could be equally or even more toxic than the original mycotoxin, and should also be taken into account [4].

All these facts make it necessary to develop analytical methods for the accurate determination of mycotoxins in different food matrices and feeds. In this sense, liquid chromatography tandem mass spectrometry (LC–MS/MS) is a powerful tool for the unique identification and quantification of analytes. Moreover, the use of high-resolution mass spectrometry (HRMS) allows the identification of novel mycotoxins and targeted/untargeted approaches for their study.

This Special Issue is dedicated to recent applications of LC–MS/MS in mycotoxin studies, including the development and validation of new analytical methods for their identification and quantification in different food matrices and feed, occurrence studies and biomonitoring of mycotoxins, and their metabolites in biological fluids.

Although most of the papers propose methods for the simultaneous determination of several mycotoxins, Bertuzzi et al. focus their work on the improvement of determination of moniliformin (MON) by adding lanthanide ions to the mobile phase during chromatographic separation [5]. MON is weakly retained in reversed-phase chromatography, making the separation difficult. The addition of La^3+^, Tb^3+^ or Eu^3+^ to the mobile phase is investigated, improving peak shape and MON separation, probably due to the formation of coordination complexes lanthanide–MON. After extraction and purification, the method is applied to the determination of MON in maize and wheat samples.

The potential of LC–MS/MS for multi-mycotoxin determination in different food matrices is shown in different contributions of this Special Issue. For instance, Guo et al. propose a modified QuEChERS (Quick, Easy, Cheap, Efficient, Rugged, and Safe) procedure for the determination of 6 *Alternaria* toxins in 56 grape samples [6]. A total of 40 samples (incidence of 71.4%) were contaminated with *Alternaria* toxins, tentoxin being the most frequently found mycotoxin (37.5%). Cannabidiol food supplements (a scarcely studied matrix) are analyzed by Narváez et al., who develop an LC–HRMS method for the quantification of 16 mycotoxins produced by major *C. sativa* fungi [7]. They report that up to six different *Fusarium* mycotoxins were found in seven of the ten analyzed samples, the most prevalent being ZEN (60%) and ENN B1 (30%). Co-occurrence was observed in four samples, including one with ENN B1, ENN A, and ENN A1. Moreover, 46 pesticides were detected after a retrospective analysis. Considering that cereals are one of the most susceptible matrices for contamination by mycotoxins, the brewing industry is an interesting field to be considered in these studies. In this context, Piacentini et al. characterize the *Fusarium* isolates from brewing barley, and the phylogenetic study shows that the majority of the strains clustered with *F. graminearum, F. poae,* and *F. avenaceum* [8]. Moreover, DON and ZEN were detected in 90.6% and 87.5% of the samples, respectively, and 86% of the samples contaminated with ZEN were above the limits set by the EU and Brazilian regulations. The same research group studied the stability of DON and ZEN during all steps of the malting and brewing processes [9]. The results show that during the malting process, DON levels decreased significantly in the steeping, germination, and malting steps (62%, 51.5%, and 68%, respectively). Considering ZEN, when the levels were compared between barley and the last step of the process, a significant decrease was observed. Another paper by Nafusa et al. reports the variation of fungal metabolites in 81 sorghum malts used to prepare Namibian traditional fermented beverages (omalodu and otombo malts) [10]. It must be highlighted that 102 metabolites were quantified in both malts (96% in omalodu malts and 93% in otombo malts). An average of 48 metabolites (including emerging mycotoxins, AF precursors, and ergot alkaloids) was quantified in otombo malts while an average of 67 metabolites was quantified in omalodu malts. Co-occurrence of EU-regulated mycotoxins, such as PAT, AFs (B1, B2, and G2), and fumonisins (FB1, FB2, and FB3) was detected in both malts with a prevalence range of 2–84%. All these studies accentuate the need to control mycotoxins in cereals intended for brewing, their resistance to food processing treatments, and their fate in the beverages.

Considering the high toxicity of mycotoxins, occurrence studies and exposure assessment in different regions of the world (especially those without a food safety regulation system) are relevant aspects, LC–MS/MS being the technique of choice for multi-mycotoxin analysis. In the framework of the first multi-center Sub-Saharan Africa Total Diet Study (SSA-TDS), 194 composite samples of cereals, tubers, legumes, vegetables, nuts and seeds, dairy, oils, beverages and miscellaneous were tested for mycotoxins [11]. The results show that populations from North Cameroon and Benin may suffer chronic and simultaneous exposure to AFB1, FB1, STC, OTA, and CIT, which are prevalent in their diet. In the same line, Ijeoma-Ekwomadu et al. investigate the variation of different *Fusarium* metabolites (including regulated, modified, and emerging mycotoxins) in 123 maize samples from various regions of South Africa [12]. The relationship between the maize producing regions, the maize type, as well as the mycotoxins, was established. The results reveal that all maize types exhibited a mixture of free, modified and emerging mycotoxins contamination across the regions with an average of 5 mycotoxins, and up to 24 out of 42 investigated *Fusarium* mycotoxins, including 1 to 3 modified forms at the same time, fumonisins (FB1, FB2, FB3, FB4, and FA1) being the most prevalent mycotoxins. Significant differences in the contamination pattern were observed between the agricultural regions and maize types. Finally, the presence of 15 mycotoxins in 120 cereal samples (including barley, maize, rice, and wheat) from Algeria is evaluated by Mahdjoubi et al. [13]. Analytical results show that 78 cereal samples (65%) were contaminated with at least one toxin, while 50% were contaminated with three to nine mycotoxins, T-2 toxin, CIT, BEA, and DON being the most frequently found. Moreover, the exposure assessment shows that the high levels of fumonisins (FB1 and FB2) in maize, and DON, ZEN, HT-2 and T-2 toxins in wheat, could represent a health risk for the average adult consumer in Algeria.

Contamination of mycotoxins in feed samples is also a matter of concern, as this is the first link in the food chain, with negative effects not only on the health of animals but also on humans consuming animal-derived products. Arroyo-Manzanares et al. carry out a survey, including 228 pig feed samples from different Spanish farms and suppliers, exploring the occurrence of 19 mycotoxins [14]. Although 97% of samples were in accordance with EU regulations, the study shows the high occurrence of emerging mycotoxins (100% samples were contaminated with ENN B and more than 90% were contaminated with BEA), as well as the high co-occurrence of different mycotoxins in the same sample (40% samples were contaminated with more than five mycotoxins). Moreover, Facorro et al. develop a methodology for the simultaneous determination of 26 mycotoxins in 97 mixed feed rations collected in 20 dairy cows farms [15]. LC–HRMS based on data-independent acquisition was employed. All unified samples analyzed showed co-occurrence of two or more mycotoxins, recurrently ZEN, FB1, and β-zearalenol, with an occurrence frequency ranging from 60% to 90%. Additional screening based on a retrospective approach enabled the identification of non-targeted mycotoxins, most of them originated from *Fusarium* fungi. The results of these studies show that, in order to ensure feed safety, emerging and modified mycotoxins, as well as the co-occurrence of different mycotoxins, should be considered as a potential risk. 

In addition to foodstuff and feed, the determination of mycotoxins in biological fluids is also one of the topics addressed in this Special Issue. Debevere et al. develop and fully-validate a UHPLC–MS/MS method for the quantitative determination in the rumen fluid of some of the most relevant mycotoxins found in maize silage [16]. Extraction recovery and matrix effects were determined in the rumen fluid with and without maize silage. Differences in extraction recovery and matrix effect between rumen fluid alone and rumen fluid with maize silage highlight the importance of using matrix-matched calibration curves for the quantification of mycotoxins in this complex sample. This method can be applied to investigate the degradation of the reported mycotoxins by rumen microbiota. Related to this topic, another aspect that is attracting great interest is biomonitoring of biological fluids as an alternative approach to assessing health risk. However, metabolic pathways have not been investigated thoroughly for all mycotoxins, and, consequently, most of the existing analytical LC–MS methods used for the assessment of human exposure focus only on the detection of parent compounds. In this context, Slobodchikova et al. perform human in vitro microsomal incubations of 17 mycotoxins in order to characterize all resulting metabolites using LC–HRMS [17]. The results allowed them to build a comprehensive LC–MS library. Moreover, the method can also screen 188 additional metabolites, including 100 metabolites reported for the first time. This contribution represents one of the most comprehensive LC–HRMS methods for mycotoxin biomonitoring or metabolism/fate studies. In other work covering this topic, Lauwers et al. propose the combination of LC–MS/MS to determine regulated and emerging mycotoxins in biological matrices of pigs and broiler chickens, with LC–HRMS to determine mycotoxin metabolites for which analytical standards are not always commercially available [18]. After in-house validation, the methods were applied to plasma, urine, feces, and/or excreta samples obtained during in vivo toxicokinetic studies with DON and ZEN in pigs, and with DON, AF B1 and OTA in broiler chickens, and during a pilot field screening study to monitor exposure to mycotoxins.

Most of the papers reported so far use solid–liquid or liquid–liquid extractions, or QuEChERS-based extractions as sample treatments. However, novel extraction methods are also proposed in this Special Issue, as in the paper of Arroyo-Manzanares et al. that explores the potential of dispersive magnetic solid-phase extraction (DMSPE) for the determination of emerging mycotoxins (ENN A, ENN A1, ENN B, ENN B1, and BEA) [19]. After its characterization, a magnetic multiwalled carbon nanotube (Fe_3_O_4_@MWCNT) composite was selected for the extraction and preconcentration of these mycotoxins in human urine samples. Analysis of the extracts was performed by LC–HRMS. Matrix-matched calibration curves were necessary, obtaining limits of quantification between 0.04 and 0.1 μg/L, relative standard deviation lower than 12%, and recoveries between 89.3% and 98.9%. 

Method validation is a very important aspect in the development of an analytical methodology. The proposed methods must assure compliance with the requirements and recommendations of mycotoxins determination in order to ensure high-quality results. Most of the published papers have carried out in-house validation. On the other hand, Bessaire et al. present the results of an intercollaborative study (including 23 entities) organized to evaluate the performance characteristics of an LC–MS/MS procedure for the determination of 12 mycotoxins in food, based on QuEChERS and immunoaffinity column cleanup [20]. Each participant analyzed 28 incurred and/or spiked blind samples composed of spices, nuts, milk powder, dried fruits, cereals, and baby food using the protocol given. The results show that the method is applicable regardless of the food, the regulated mycotoxin, and the concentration level (even at low regulated levels for foods intended for infants and young children), therefore being an excellent candidate for future standardization. 

## References

[1] Rapid Alert System for Food and Feed (RASFF). https://ec.europa.eu/food/safety/rasff_en.

[2] European Commission Mycotoxins. https://ec.europa.eu/food/safety/chemical_safety/contaminants/catalogue/mycotoxins_en.

[3] Gruber-Dorninger C., Novak B., Nagl V., Berthiller F. (2017). Emerging mycotoxins: Beyond traditionally determined food contaminants. J. Agric. Food Chem..

[4] Freire L., Sant’Ana A.S. (2018). Modified mycotoxins: An updated review on their formation, detection, occurrence, and toxic effects. Food Chem. Toxicol..

[5] Bertuzzi T., Rastelli S., Mulazzi A., Pietri A. (2019). LC-MS/MS and LC-UV Determination of moniliformin by adding lanthanide ions to the mobile phase. Toxins.

[6] Guo W., Fan K., Nie D., Meng J., Huang Q., Yang J., Shen Y., Tangni E.K., Zhao Z., Wu Y. (2019). Development of a QuEChERS-based UHPLC-MS/MS method for simultaneous determination of six *Alternaria* toxins in grapes. Toxins.

[7] Narváez A., Rodríguez-Carrasco Y., Castaldo L., Izzo L., Ritieni A. (2020). Ultra-high-performance liquid chromatography coupled with quadrupole Orbitrap high-resolution mass spectrometry for multi-residue analysis of mycotoxins and pesticides in botanical nutraceuticals. Toxins.

[8] Piacentini K.C., Rocha L.O., Savi G.D., Carnielli-Queiroz L., De Carvalho Fontes L., Correa B. (2019). Assessment of toxigenic *Fusarium* species and their mycotoxins in brewing barley grains. Toxins.

[9] Piacentini K.C., Běláková S., Benešová K., Pernica M., Savi G.D., Rocha L.O., Hartman I., Čáslavský J., Corrêa B. (2019). *Fusarium* mycotoxins stability during the malting and brewing processes. Toxins.

[10] Nafuka S.N., Misihairabgwi J.M., Bock R., Ishola A., Sulyok M., Krska R. (2019). Variation of fungal metabolites in sorghum malts used to prepare Namibian traditional fermented beverages *Omalodu* and *Otombo*. Toxins.

[11] Ingenbleek L., Sulyok M., Adegboye A., Hossou S.E., Koné A.Z., Oyedele A.D., Kisito C.S.K.J., Dembélé Y.K., Eyangoh S., Verger P. (2019). Regional Sub-Saharan Africa total diet study in Benin, Cameroon, Mali and Nigeria reveals the presence of 164 mycotoxins and other secondary metabolites in foods. Toxins.

[12] Ekwomadu T.I., Dada T.A., Nleya N., Gopane R., Sulyok M., Mwanza M. (2020). Variation of Fusarium free, masked, and emerging mycotoxin metabolites in maize from agriculture regions of South Africa. Toxins.

[13] Mahdjoubi C.K., Arroyo-Manzanares N., Hamini-Kadar N., García-Campaña A.M., Mebrouk K., Gámiz-Gracia L. (2020). Multi-mycotoxin occurrence and exposure assessment approach in foodstuffs from Algeria. Toxins.

[14] Arroyo-Manzanares N., Rodríguez-Estévez V., Arenas-Fernández P., García-Campaña A.M., Gámiz-Gracia L. (2019). Occurrence of mycotoxins in swine feeding from Spain. Toxins.

[15] Facorro R., Llompart M., Dagnac T. (2020). Combined (d)SPE-QuEChERS extraction of mycotoxins in mixed feed rations and analysis by high performance liquid chromatography-high-resolution mass spectrometry. Toxins.

[16] Debevere S., De Baere S., Haesaert G., Rychlik M., Fievez V., Croubels S. (2019). Development of an UPLC-MS/MS method for the analysis of mycotoxins in rumen fluid with and without maize silage emphasizes the importance of using matrix-matched calibration. Toxins.

[17] Slobodchikova I., Sivakumar R., Rahman M.S., Vuckovic D. (2019). Characterization of Phase I and glucuronide Phase II metabolites of 17 mycotoxins using liquid chromatography—High-resolution mass spectrometry. Toxins.

[18] Lauwers M., De Baere S., Letor B., Rychlik M., Croubels S., Devreese M. (2019). Multi LC-MS/MS and LC-HRMS methods for determination of 24 mycotoxins including major Phase I and II biomarker metabolites in biological matrices from pigs and broiler chickens. Toxins.

[19] Arroyo-Manzanares N., Peñalver-Soler R., Campillo N., Viñas P. (2020). dispersive solid-phase extraction using magnetic carbon nanotube composite for the determination of emergent mycotoxins in urine samples. Toxins.

[20] Bessaire T., Mujahid C., Mottier P., Desmarchelier A. (2019). Multiple mycotoxins determination in food by LC-MS/MS: An international collaborative study. Toxins.

